# Complete genome sequence of bile-isolated *Enterococcus avium* strain 352

**DOI:** 10.1186/s13099-019-0294-9

**Published:** 2019-04-24

**Authors:** Tao Yu, Lixiang Li, Qilin Zhao, Peng Wang, Xiuli Zuo

**Affiliations:** 1 Department of Gastroenterology, Qilu Hospital, Shandong University, 107 Wenhuaxi Road, Jinan, 250012 Shandong People’s Republic of China; 2 Laboratory of Translational Gastroenterology, Qilu Hospital, Shandong University, Jinan, 250012 People’s Republic of China; 3 High School Attached To Shandong Normal University, Jinan, 250012 People’s Republic of China

**Keywords:** *E. avium*, Virulence factors, Bile adaption

## Abstract

**Background:**

*Enterococcus avium* is a Gram-positive pathogenic bacterium belonging to the family *Enterobacteriaceae. E. avium* can cause bacteremia, peritonitis, and intracranial suppurative infection. However, the mechanism of its pathogenesis and its adaptation to a special niche is still unclear.

**Results:**

In this study, the *E. avium* strain 352 was isolated from human bile and whole genome sequencing was performed. The *E. avium* strain 352 consists of a circular 4,794,392 bp chromosome as well as an 87,705 bp plasmid. The GC content of the chromosome is 38.98%. There are 4905 and 99 protein coding sequences in the chromosome and the plasmid, respectively. The genome of the *E. avium* strain 352 contains number of genes reported to be associated with bile adaption, including bsh, *sbcC*, *mutS*, *nifI*, galU, and *hupB*. There are also several virulence-associated genes including *esp*, *fss1*, *fss3*, *ecbA*, *bsh*, *lap*, *clpC*, *clpE*, and *clpP*.

**Conclusions:**

This study demonstrates the presence of various virulence factors of the *E. avium* strain 352, which has the potential to cause infections. Moreover, the genes involved in bile adaption might contribute to its ability to live in bile. Further comparative genomic studies would help to elucidate the evolution of pathogenesis of *E. avium*.

## Background

*Enterococcus avium* is a Gram-positive bacterium of the genus *Enterococcus* and is most commonly found in birds. *E. avium* is also a cause of infectious diseases in humans including bacteremia, peritonitis, intracranial suppurative infection and osteomyelitis [1–5]. It was reported that *E. avium* is responsible for approximately 1% of infections in humans [3]. However, there is not much known about the mechanism of its pathogenesis.

*Enterococcus avium* was isolated from blood samples, fecal samples, spinal cords, jeotgals (a Korean fermented seafood), and scallop solutions [1, 3, 6, 7]. Thus, *E. avium* can adapt to various environments and this might be an important factor for its survival in humans and for subsequent infections. Currently, there are 8 draft genomes of *E. avium* accessible on NCBI databases. However, no studies have analyzed these genomes for the niche adaptation of *E. avium*.

Here, we report the first whole genome sequence of *E. avium*. We also analyzed the virulence-associated genes and bile stress adaptation mechanism of the *E. avium strain* 352.

## Methods

### Strain isolation and characterization

The *E. avium* strain 352 was isolated from a bile sample of a cholelithiasis patient. This strain was cultivated on blood plate agar under anaerobic conditions at 37 °C for 24 h. This strain was identified by 16S rRNA sequencing using the following primers including 27F (5′-AGAGTTTGATCCTGGCTCAG-3′) and 1492R (5′-GGTTACCTTGTTACGACTT-3′). The PCR products were subsequently sequenced, and these sequences were compared against the 16S rRNA bacteria sequence database using BLAST from the NCBI website.

### Genome sequencing and de novo assembly

The bacterial genomic DNA was extracted from overnight culture of the *E. avium* 352 using the Bacteria DNA Kit (OMEGA Bio-Tek Inc., Norcross, GA, USA) according to the manufacturer’s instructions, and quality control was subsequently carried out using TBS-380 fluorometer (Turner BioSystems Inc., Sunnyvale, CA). Then, high qualified DNA sample (OD260/280 = 1.8–2.0, > 6 μg) was utilized to construct a fragment library.

Genomic DNA (above 3 μg) was subjected to whole genome sequencing on an Illumina HiSeq Sequencer (PE150 mode) according to the sequencing protocol. Raw sequencing data was generated by Illumina base calling software CASAVA v1.8.2 (Illumina Inc. San Diego, CA, USA). Contamination reads, such as ones containing adaptors or primers were identified by Trimmomatic with default parameters. Clean data obtained by above quality control processes were used to do further analysis. Meanwhile, the whole-genome sequencing of *E. avium* 352 was also carried out on the single molecule real-time by the PacBio RS Platform (Pacific Biosciences of California, Inc., Menlo Park, CA, USA). A 20 K template library was generated and sequenced using standard methods.

The Illumina data were used to evaluate the complexity of the genome and correct the PacBio long reads. First, we used ABySS to peform genome assembly with multiple-Kmer parameters and obtained optimal results for the assembly [8]. Second, canu (https://github.com/marbl/canu) was used to assemble the PacBio corrected long reads [9]. Finally, GapCloser software was subsequently applied to fill the remaining local inner gaps and correct the single base polymorphism for the final assembly results [10].

Gene annotation was determined by Annotation NCBI Prokaryotic Genome Annotation Pipeline [11]. Ribosomal RNA genes were detected by RNAmer 1.2 [12] and tRNA genes were recognized via tRNAscan SE v. 2.0 [13]. The circular genomic map was produced using CGView Server [14].

Phylogenetic analysis is based on orthologous genes. First, orthologous gene families were identified by the ORTHOMCL v2.0 program (reciprocal all-by-all BLASTP analysis) with an E-value of 10^−5^ [15]. Second, multiple alignments were generated with the MUSCLE v3.8.31 program, and the alignments were examined visually [16]. Third, the Maximum-likelihood (ML) methods were performed for the phylogenetic analyses using PhyML 3.0, and the model GTR + G was selected for ML analyses with 500 bootstrap replicates to calculate the bootstrap values [17]. The strains used for phylogenetic tree analysis included the *E. avium* strain ATCC 14025 (GCA000406965.1), the *E. faecalis* strain ATCC 19433 (GCA000392875.1), the *E. faecium* strain DO (GCA000174395.2), the *E. gilvus* strain ATCC BAA-350 (GCA_000394615.1), the *E. pseudoavium* strain CBA7133 (GCA 003386455.1), the *E. sulfureus* strain ATCC 49903 (GCA000407025.1), the *E. raffinosus* strain ATCC 49464 (GCA000393895.1), the *E. gallinarum* strain FDAARGOS_163 (GCA001558875.2), and the *Vagococcus fluvialis* strain DSM 5731 (GCA003337315.1). The putative virulence related genes were identified based on the whole genome of the *E. avium* strain 352 using the VFDB [18].

### Quality assurance

A single colony of the *E. avium* strain 352 was repeatedly transferred to fresh brain heart infusion (BHI) medium to obtain pure cultures. Before DNA extraction, the identity of the strain was verified through 16S rRNA gene sequencing. After the genome sequence was obtained, the 16S rDNA gene was extracted from the genome using the RNAmmer 1.2 server and then confirmed through a BLAST search of the 16S rRNA gene against the NCBI microbial 16S database.

## Results and discussion

### General genome features of the *E. avium* strain 352

**Fig. 1:**
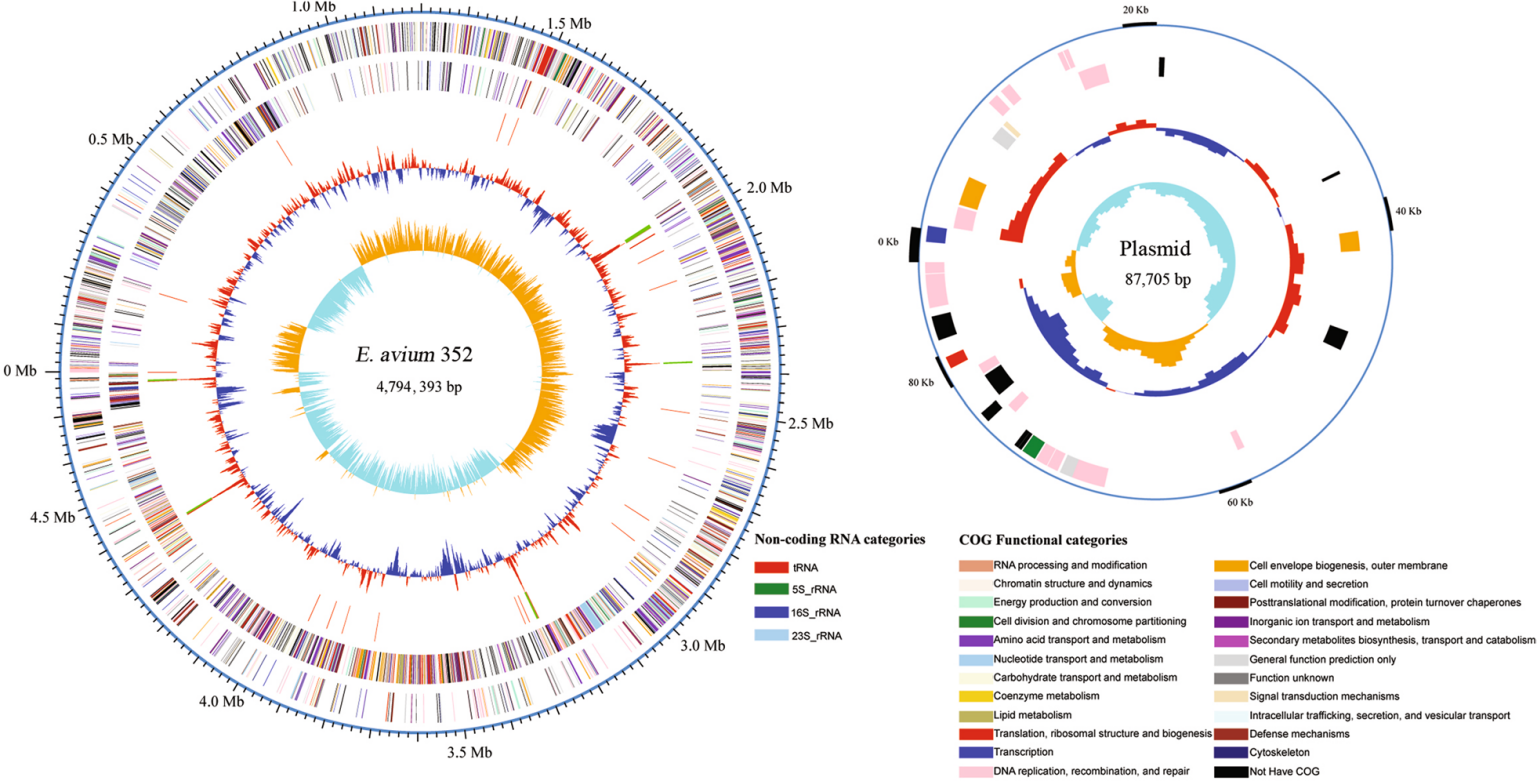
Genome map of *E. avium* 352. Circles from the outside to inside showing: (1) DNA coordinates; (2, 3) function-based color-coded mapping of the CDSs predicted on the forward and reverse strands. Functions are color-coding; (4) tRNA genes and rRNA genes; (5) GC plot showing regions above the average (red) and below (blue); (6) GC skew

Total of 46,188,978 raw reads were obtained by Illumina HiSeq Sequencer, and 45,357,196 high quality reads were generated after quality control processes. In addition, 168,754 (1.26 Gb) high-quality reads with an average read length of 7500 bp and a 259-fold coverage were generated by PacBio sequencer. These sequences were used to assemble the genome of the *E. avium* strain 352 and we obtained a circular chromosome without gap. The complete genome is 4.79 Mb in size with a plasmid of 87.7 kb (Fig. 1) and the mean G + C content is 38.98%. This genome contains 4905 predicted genes as well as 18 rRNA and 68 tRNA genes, while there were 99 predicted genes in the plasmid.

### Phylogenetic analysis

**Fig. 2:**
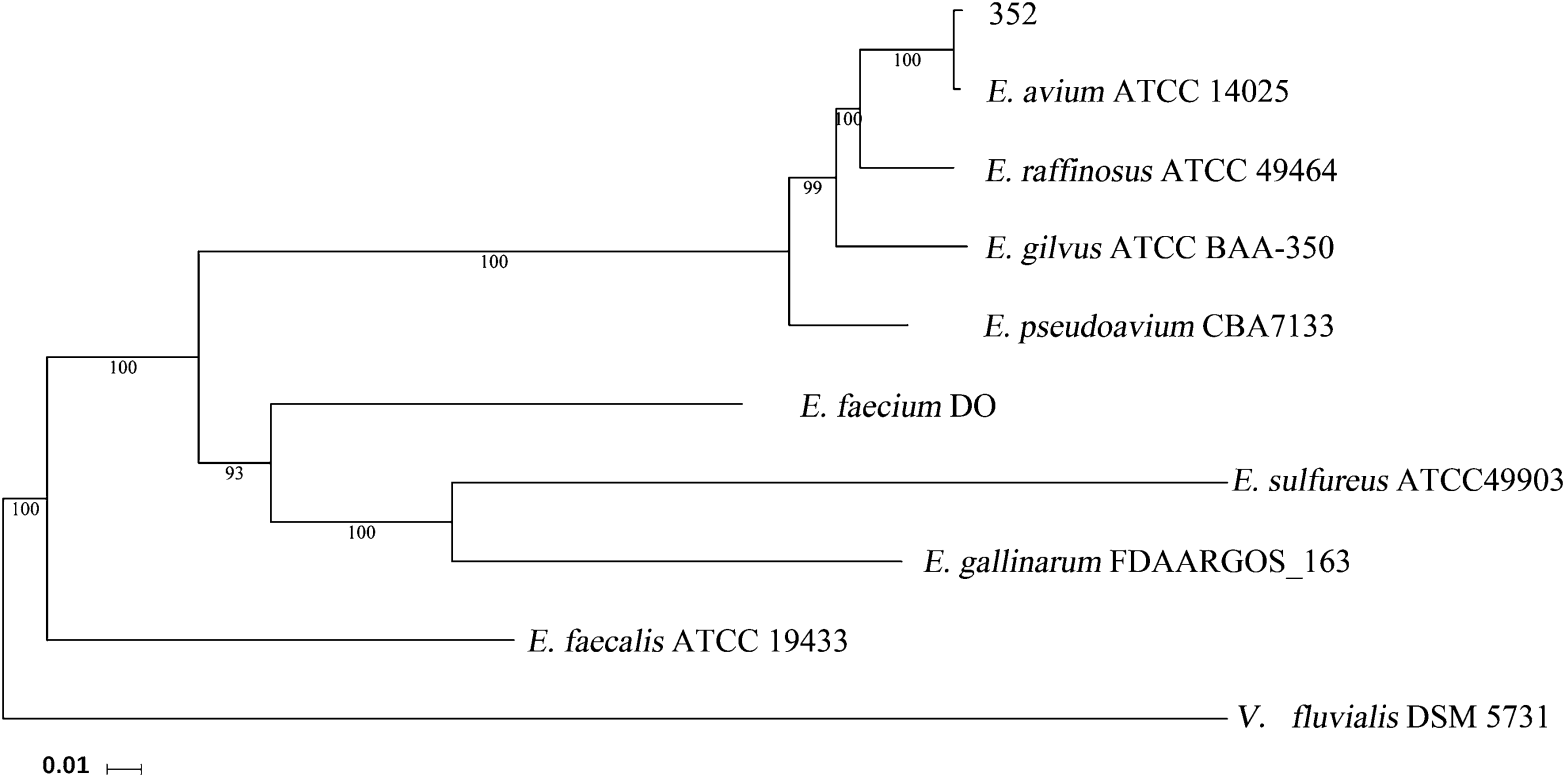
Phylogenetic analysis of *E. avium* 352

The 16S rRNA gene sequence verified the taxonomic status of the *E. avium* strain 352 (data not shown). To further elucidate the phylogenetic relationships, whole genome DNA-sequence-based phylogenetic analysis was carried out (Fig. 2). The genome of a highly related and similar type of *E. avium* strain, *E. avium strain* ATCC 14025, was selected as standard. The dendrogram of phylogenetic trees illustrated that the *E. avium* strain 352 was most closely related to the *E. avium* strain ATCC 14025.

### Identification of genes related to bile stress

Bile salts have potent antimicrobial activity via damaging membranes and DNA. Thus, bacteria must have intrinsic adapted mechanisms to survive in bile and subsequently cause biliary tract infections [19]. Genomic analysis of the *E. avium* strain 352 showed the presence of numerous genes that may determine its bile resistance properties (Table 1). The presence of the genes *sbcC*, *mutS* and *nifI* involved in bile resistance in Gram-positive bacteria was identified [19]. It is interesting that there were two *bsh* genes encoding bile salt hydrolase with a protein sequence identity of 92.9% in the genome of the *E. avium* strain 352. This result indicated that the BSH might be play an important role in niche-specific adaptation for bile [20]. There were also some genes, including *galU* and *hupB*, involved in bile resistance in Gram-negative bacteria [19]. Further studies are needed to verify its genetic properties and evolution traits.

**Table 1:** Putative genes for bile adaptation in *E. avium* 352

Gene name	Fuction/putative fuction
*hupB*	HU family DNA-binding protein
*galU*	UDP-glucose-pyrophosphorylase
*sbcC*	Exonuclease SbcC
*mutS*	DNA mismatch repair
*bsh1*	Bile salt hydrolase
*bsh*2	Bile salt hydrolase
*nifI*	Pyruvate: ferredoxin oxidoreductase

### Analysis of virulence associated genes

Further screening the genome of the *E. avium* strain 352 for putative virulence-associated genes was conducted by aligning gene sequences to the virulence factor database (Table 2). There are surface protein encoded genes including *esp*, *fss1* and *fss3*. The *E. avium* strain 352 also contains the conservative heat shock protein genes *clpC*, *clpE*, and *clpP* [21]. The *ecbA* gene encoding a collagen binding MSCRAMM (acronym for microbial surface components recognizing adhesive matrix molecules) and gene *lap* encoding a listeria adhesion protein were found in the genome and might be contribute to adherence to the host tissue [22, 23]. The *bsh* gene encoding a bile salt hydrolase was also a virulence related factor in *Listeria monocytogenes* [24]. The clinical significance of this finding warrants further investigation.

**Table 2:** Putative virulence associated genes in *E. avium* 352 predicted by VFDB

Gene name	Function/putative function	Score	E value
*esp*	Enterococcal surface protein	3907	0
*fss*3	*Enterococcus faecalis* surface protein Fss3	3749	0
*ecbA*	Collagen binding MSCRAMM	3416	0
*bsh*	Bile salt hydrolase	274	6e−70
*lap*	Listeria adhesion protein	129	2e−26
*clpE*	ATP-dependent protease	127	9e−26
*clpP*	ATP-dependent c1p protease peoteolytic subunit	117	8e−23
*fss1*	*Enterococcus faecalis* surface protein Fss1	107	8e−20
*clpC*	Endopeptidase C1p ATP-binding chain C	76	3e−10
